# Airway Dysfunction in Obesity: Response to Voluntary Restoration of End Expiratory Lung Volume

**DOI:** 10.1371/journal.pone.0088015

**Published:** 2014-02-04

**Authors:** Beno W. Oppenheimer, Kenneth I. Berger, Leopoldo N. Segal, Alexandra Stabile, Katherine D. Coles, Manish Parikh, Roberta M. Goldring

**Affiliations:** 1 André Cournand Pulmonary Physiology Laboratory, Division of Pulmonary, Critical Care and Sleep, Department of Medicine, Bellevue Hospital/New York University School of Medicine, New York, New York, United States of America; 2 Bellevue Hospital Bariatric Center, Department of Surgery, New York University School of Medicine, New York, New York, United States of America; Mayo Clinic College of Medicine, United States of America

## Abstract

**Introduction:**

Abnormality in distal lung function may occur in obesity due to reduction in resting lung volume; however, airway inflammation, vascular congestion and/or concomitant intrinsic airway disease may also be present. The goal of this study is to 1) describe the phenotype of lung function in obese subjects utilizing spirometry, plethysmography and oscillometry; and 2) evaluate residual abnormality when the effect of mass loading is removed by voluntary elevation of end expiratory lung volume (EELV) to predicted FRC.

**Methods:**

100 non-smoking obese subjects without cardio-pulmonary disease and with normal airflow on spirometry underwent impulse oscillometry (IOS) at baseline and at the elevated EELV.

**Results:**

FRC and ERV were reduced (44±22, 62±14% predicted) with normal RV/TLC (29±9%). IOS demonstrated elevated resistance at 20 Hz (R_20_, 4.65±1.07 cmH_2_O/L/s); however, specific conductance was normal (0.14±0.04). Resistance at 5–20 Hz (R_5−20_, 1.86±1.11 cmH_2_O/L/s) and reactance at 5 Hz (X_5_, −2.70±1.44 cmH_2_O/L/s) were abnormal. During elevation of EELV, IOS abnormalities reversed to or towards normal. Residual abnormality in R_5−20_ was observed in some subjects despite elevation of EELV (1.16±0.8 cmH_2_O/L/s). R_5−20_ responded to bronchodilator at baseline but not during elevation of EELV.

**Conclusions:**

This study describes the phenotype of lung dysfunction in obesity as reduction in FRC with airway narrowing, distal respiratory dysfunction and bronchodilator responsiveness. When R_5−20_ normalized during voluntary inflation, mass loading was considered the predominant mechanism. In contrast, when residual abnormality in R_5−20_ was demonstrable despite return of EELV to predicted FRC, mechanisms for airway dysfunction in addition to mass loading could be invoked.

## Introduction

Recently there has been increased awareness of the interaction between obesity and intrinsic airway diseases such as asthma. However, objective assessment of lung function in obese patients is confounded by functional abnormalities that are attributable to increased body weight. Airway dysfunction will occur in obese subjects due to reduction in resting lung volume with associated airway compression; however, airway inflammation, vascular congestion and/or concomitant intrinsic airway disease may also be present.[1]–[5] Distinguishing the contribution of airway compression due to mass loading from these other mechanisms [6], [7] is clinically important and may not be identified by standard physiologic testing.

In obesity, mass loading shifts the balance of forces between chest wall/abdomen and lung and produces a decrease in functional residual capacity (FRC). [3], [8] The resulting decreased airway diameter is manifest as reduction in expiratory reserve volume (ERV). [8] Assessment of distal airways has been elusive to traditional testing techniques due to their large aggregate cross-sectional area and minimal contribution to total airway resistance.[9]–[11] Forced oscillation testing has demonstrated elevated resistance associated with reduction of resting lung volumes as demonstrated by measurement of airway conductance.[12]–[15] Additionally, frequency dependence of resistance is observed despite normal ratio of forced expiratory volume in 1 second (FEV_1_) to vital capacity (VC), [12], [16] suggesting dysfunction in airways more distal than those assessed by spirometry.[17]–[19].

The present study utilizes forced oscillation to further investigate the interaction between lung volume and airway function in healthy obese subjects. Previous studies from this laboratory have demonstrated reversal of oscillometric abnormalities following weight loss but could not attribute it to relief of mass loading alone since obesity associated airway inflammation and vascular congestion could also have improved. [20] Therefore, the present study evaluates lung function prior to weight loss, before and during a voluntary inflation maneuver targeted to acutely relieve airway compression by restoring end-expiratory volume (EELV) to predicted FRC. This maneuver should reverse the effect of mass loading on the lung allowing detection of functional abnormalities unrelated to mass loading per se.

## Methods

### Subjects

The present study analyzed data from 100 obese subjects (BMI >30 kg/m^2^) referred from the Bariatric Center to the André Cournand Pulmonary Physiology Laboratory in Bellevue Hospital for evaluation prior to weight reduction surgery. Subjects underwent spirometry, plethysmography and impulse oscillometry (IOS). Records were reviewed to determine symptoms and medical history. Subjects were excluded for history of smoking or history of pulmonary or cardiac disease. Since abnormal spirometric flow rates are not a part of the obesity phenotype [8], subjects were included based on presence of normal spirometry, defined as FEV_1_ and VC ≥80% of predicted and FEV_1_/VC ≥77%. [21].

### Spirometry and Lung Volumes

Pulmonary function testing (Vmax; Sensor-Medics; Yorba Linda, CA) was performed in accord with published standards. [22], [23] The maximal negative inspiratory pressure was measured at FRC. FRC was determined (n = 93) by plethysmography and, when not physically feasible, by nitrogen washout.

### Impulse Oscillometry

**Figure 1:**
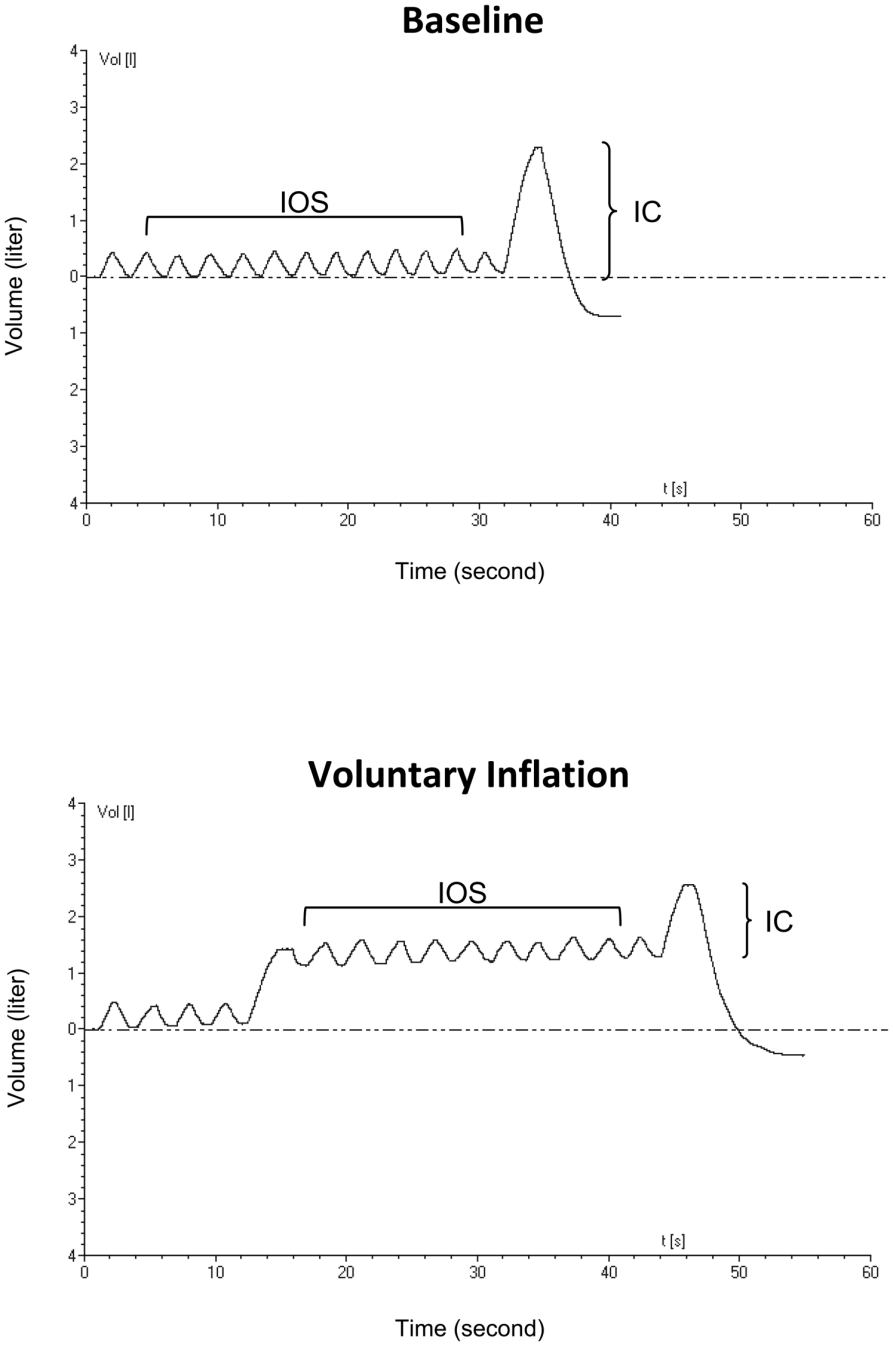
Image obtained from the oscillometry system illustrating measurement at baseline FRC (top panel) and following voluntary inflation targeted to achieve an end expiratory lung volume similar to the predicted FRC (bottom panel).

Impulse oscillometry (IOS) was performed utilizing the Jaeger Impulse Oscillation System (Jaeger USA; Yorba Linda, CA) under 2 conditions (Figure 1). First, measurements were obtained at baseline FRC during tidal breathing in the seated position with support of the cheeks. At the end of each measurement, inspiratory capacity (IC) was measured. Second, subjects performed a voluntary inflation until reaching a target end expiratory lung volume (EELV) close to predicted FRC. At the elevated EELV, subjects were instructed to breathe with tidal volume and frequency similar to baseline. At the end of the maneuver, IC was again measured. The EELV during voluntary inflation was determined by adding the change in IC (baseline to voluntary inflation) to the baseline FRC and was expressed as a percentage of the predicted FRC. A minimum of 3 trials of 30 seconds duration was performed at each EELV. Trials with constant tidal volume and stable EELV were analyzed. Reproducibility between trials (variability <10%) and coherence >0.7 at 5 Hz and >0.85 at 10 Hz were required. [24], [25] Bronchodilator responsiveness was assessed following inhaled albuterol. IOS data were analyzed only when the pre and post bronchodilator EELV agreed within 10%.

Parameters reported included resistance at oscillation frequencies of 5 Hz (R_5_) and 20 Hz (R_20_), frequency dependence of resistance calculated as the difference between resistance at 5 and 20 Hz (R_5−20_), reactance at 5 Hz (X_5_), resonant frequency (F_res_) and reactance area (AX). For the purpose of evaluating properties of the respiratory system, assumptions were made based on the models of DuBois et al., Otis et al. and Mead. R_5_ and R_20_ was assumed to reflect respiratory system resistance at 5 and 20 Hz respectively, R_5−20_ and AX reflect non-uniform distribution of airflow in distal airways, and X_5_ was assumed to reflect dynamic elastance. In accord with these assumptions, prior observations from our laboratory have demonstrated that R_20_ correlates with measurements of airway resistance obtained with esophageal manometry, R_5−20_ correlates with frequency dependence of compliance, and X_5_ correlates with dynamic lung compliance.[17]–[19], [26]–[29] Specific conductance was calculated for R_20_ (SG_rs_ @ 20 Hz) by dividing 1/R_20_ by the EELV at which it was measured.

Data are presented as raw data and are compared to published limits of normal.[17], [30]–[35] Normative data for SG_rs_ @ 20 Hz were compared to the mean value ±1.65 SD (0.12±0.04 cmH_2_O/L/s) calculated from 46 asymptomatic, non-obese (BMI ≤27 kg/m^2^) non-smoking subjects without lung disease and in whom spirometry, lung volumes and IOS were within normal limits. These subjects had characteristics (age range 20–59 years, height 1.5–1.9 meters, 75% female) similar to the obese subjects evaluated in this study.

### Statistical Analysis

Data were summarized as mean ± standard deviation (SD) or standard error (SE). Differences between groups were assessed utilizing a Student’s t test or Mann-Whitney U test. Correlation coefficients were derived from linear regressions. Analyses were performed utilizing SPSS for Windows version 17.0.

### Ethics Statement

This study was approved by the institutional review boards of the NYU School of Medicine and Bellevue Hospital (protocol #09-0756 and 09-0354). A consent process was not deemed necessary by the board, as the tests performed in this study are part of routine pulmonary function testing obtained in these patients prior to bariatric surgery.

The study was conducted in accord with the principles expressed in the Declaration of Helsinki.

## Results

Table 1 presents clinical characteristics of the 100 subjects. The majority were female. Mean age was 42±12 years and mean BMI was 44±6 kg/m^2^. Symptoms were reported by 43% of subjects; the predominant being dyspnea reported in 37% despite no history of cardio-pulmonary disease.

**Table 1:** Clinical Characteristics (n = 100).

Characteristics	Data
Age, yr	42±12*
Females	90%
Anthropometric Data	
Height (m)	1.60±0.09
Weight (kg)	113±20
BMI (kg/m^2^)	44±6
Waist Circumference (in)	
Men (n = 9)	55±5
Women (n = 69)	50±7
Associated Disease	
Hyperlipidemia	25%
Hypertension	34%
Sleep Apnea	10%
Diabetes	19%
Symptoms†	
Cough	9%
Dyspnea	37%
Wheeze	4%
Chest tightness	12%

*Mean ± SD.

† Multiple symptoms may be present.

### Lung Function Phenotype

**Figure 2:**
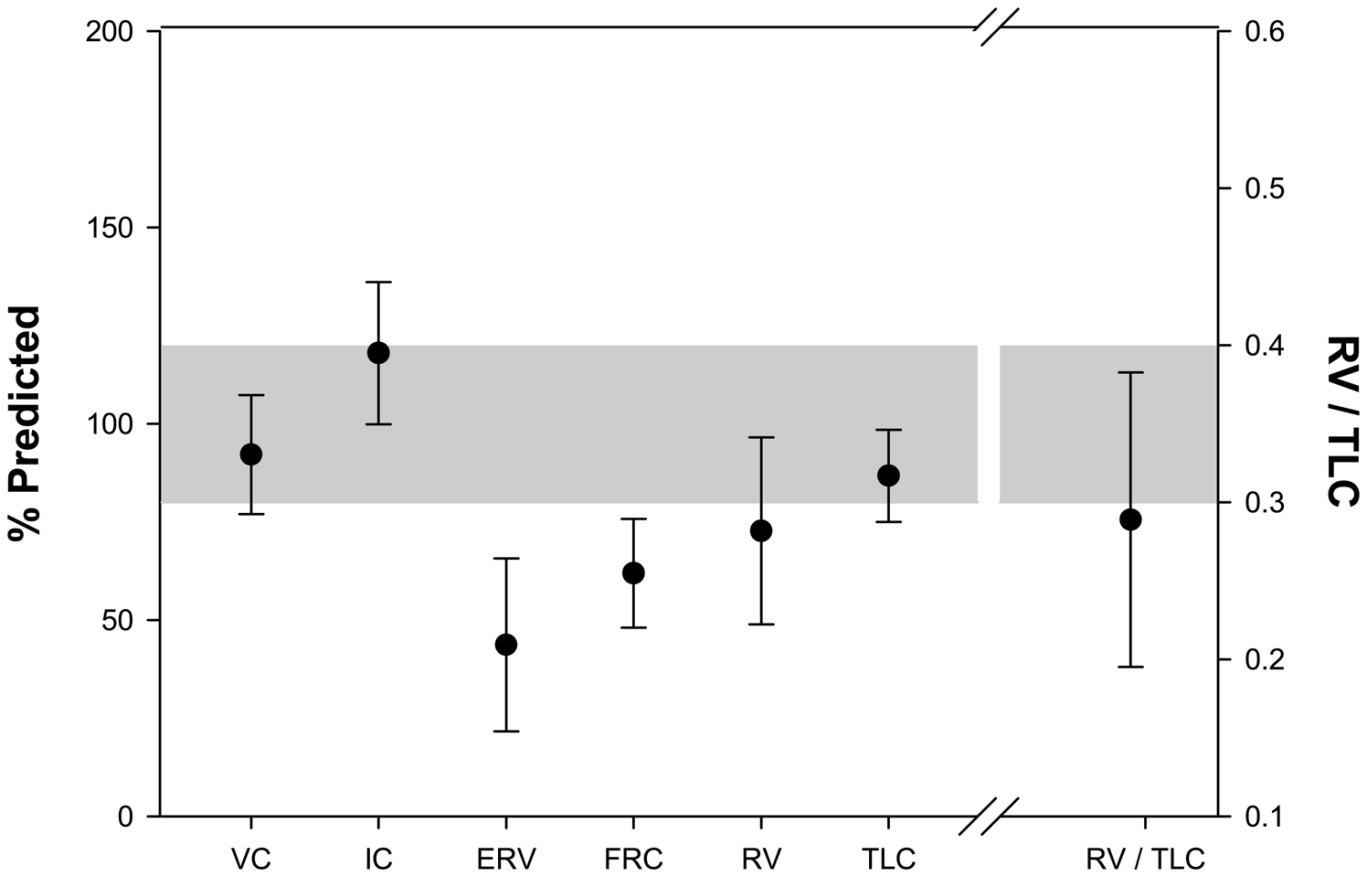
Baseline lung volumes are illustrated. Data are expressed as mean ± SD. Shaded area represents normal range.

The results of lung volume parameters are illustrated in Figure 2. The mean value for VC was normal (94±15% predicted). VC remained normal despite marked reduction in ERV in all subjects (44±22% predicted) associated with an increase in IC above the normal range (118±18% predicted). FRC and residual volume (RV) were reduced (62±14 and 73±24% predicted, respectively). There was no evidence of air trapping as indicated by normal RV to total lung capacity ratio (TLC) ratio (29±9%). Although the mean value for TLC was normal (87±12% predicted), 25 subjects demonstrated TLC<80% predicted. Reduced TLC occurred when IC failed to increase above the normal range.

**Figure 3:**
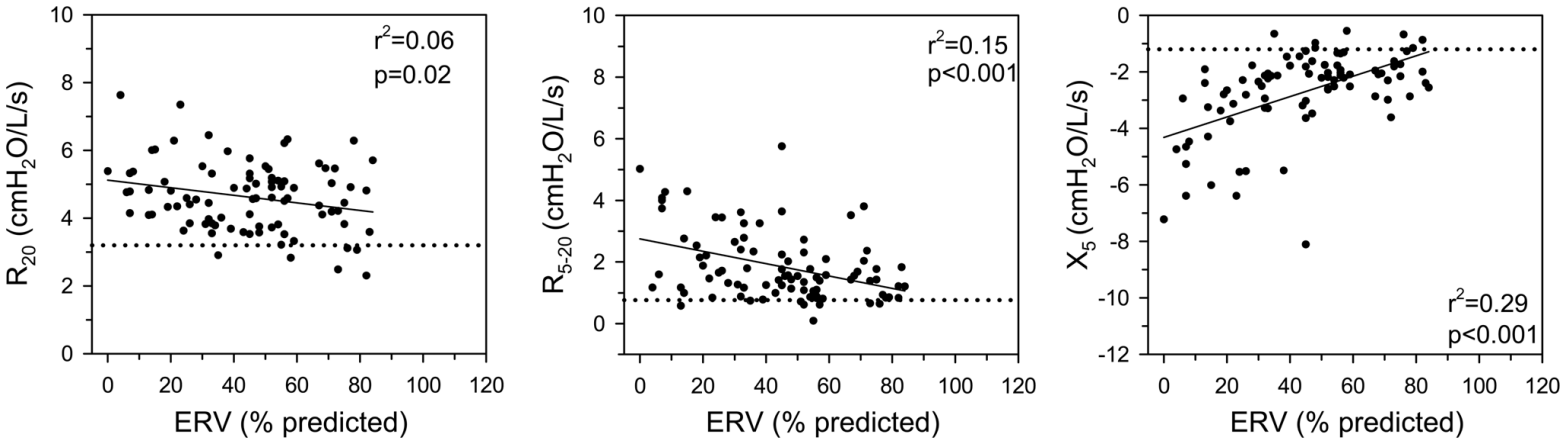
Individual values for R_20_, R_5−20_, and X_5_ are related to ERV. Dotted line represents the upper limit of normal for each parameter.

Spirometry and oscillometry data are shown in Table 2. FEV_1_/VC was within normal limits in all subjects (by design). However, mean values for all oscillometry parameters were abnormal. Analysis of individual data revealed abnormalities in >90% of these obese subjects. IOS parameters bore minimal relationship to FRC and to body size over the range of BMI from 33 to 58 kg/m^2^ (r^2^≤0.08 for all IOS parameters; data not shown). In contrast, Figure 3 illustrates a significant correlation between both R_5−20_ and X_5_ to reduction in ERV (r^2^ = 0.15, p = 0.0002 and r^2^ = 0.29, p<0.0001 respectively).

**Table 2:** Results of spirometry and oscillometry.

	Mean ± SD	Upper Limit of Normal
**Spirometry (n = 100)**		
FVC (% predicted)	93±15	
FEV_1_ (% predicted)	90±14	
FEV_1_/FVC (%)	83±4	
Max. insp. pressure (cmH_2_O)	−85±32	
**Oscillometry (n = 94)**		
Resistance parameters		
R_5_ (cmH_2_O/L/s)	6.51±1.71	<3.96
R_20_ (cmH_2_O/L/s)	4.65±1.07	<3.20
Frequency dependent parameters		
R_5–20_ (cmH_2_O/L/s)	1.86±1.11	<0.76
AX (cmH_2_O/L)	18.47±13.92	<3.60
Reactance parameters		
X_5_ (cmH_2_O/L/s)	−2.70±1.44	> −1.2
Resonant Frequency (Hz)	20.11±4.69	<12

### Voluntary Inflation

IOS was repeated at an elevated EELV targeted to achieve a lung volume similar to the predicted FRC; data are analyzed for 71 subjects with acceptable maneuvers (see methods). Lung inflation averaged 0.90±0.41 liters, which restored EELV on average to 93±3% of predicted FRC; 50/71 subjects achieved an EELV ≥80% of predicted FRC.

**Figure 4:**
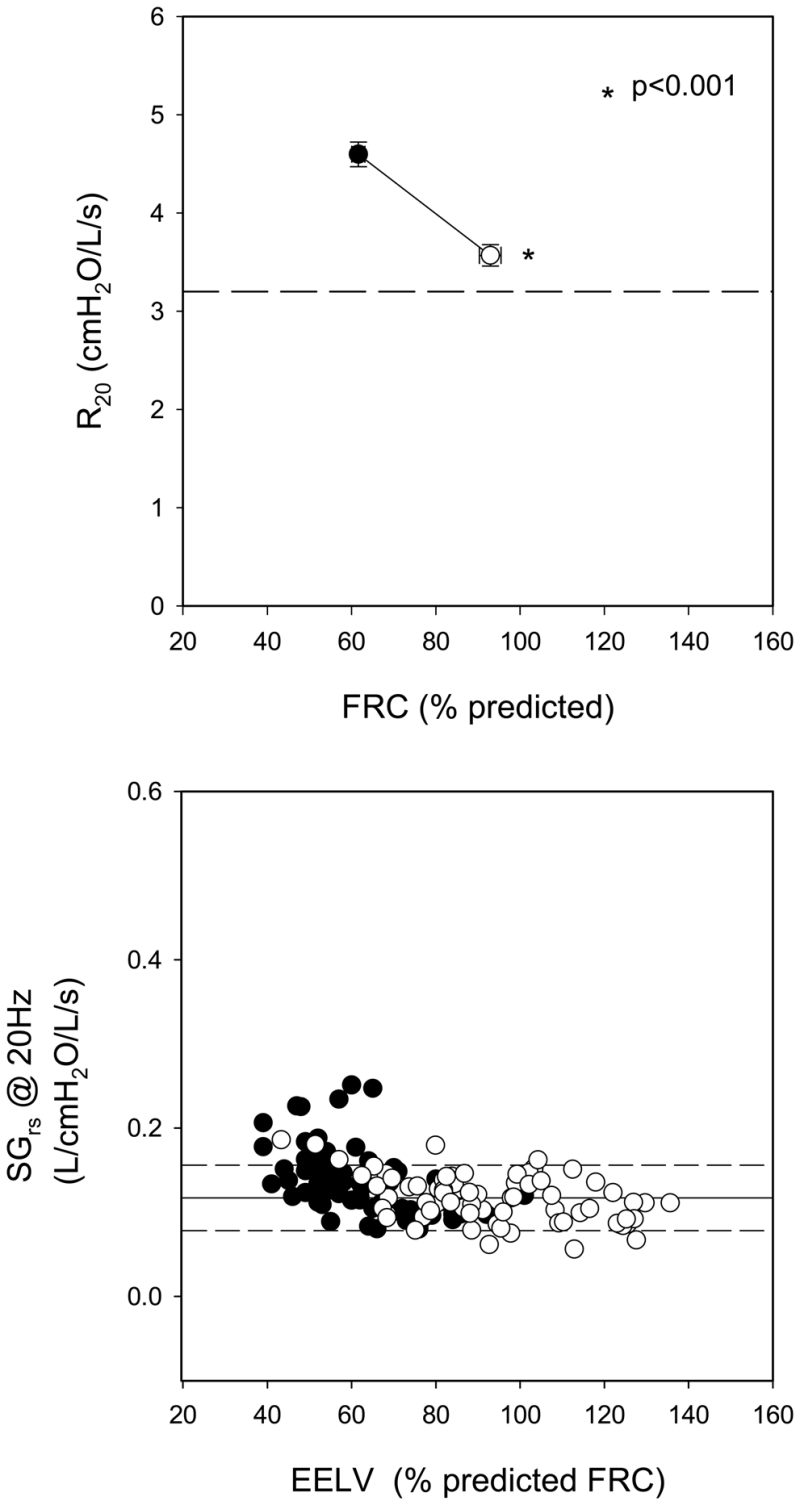
Top Panel: R_20_ at baseline and during voluntary inflation. Data are mean ± SE. Dotted line represents the upper limit of normal. Bottom Panel: Individual values for specific conductance at 20 Hz (SG_rs_ @ 20 Hz) plotted as a function of the absolute EELV at which it was obtained. EELV is expressed as % predicted FRC. Dotted and solid lines represent the mean ±1.65 SD calculated from data obtained in normal subjects. (• = baseline ○ = voluntary inflation).

The effect of voluntary inflation on R_20_ is illustrated in Figure 4. R_20_ significantly decreased towards normal (4.64±0.12 to 3.57±0.11 cmH_2_O/l/s, p<0.001; top panel). When R_20_ did not correct to normal it was associated with failure of EELV to reach predicted FRC. The lower panel re-expresses R_20_ as specific conductance (SG_rs_ @ 20 Hz) for each individual. Despite high resistance, specific conductance was normal at baseline (closed symbols) and remained unchanged (open symbols) during voluntary inflation (0.14±0.04 and 0.12±0.03 L/cmH_2_O/L/s, respectively, NS).

**Figure 5:**
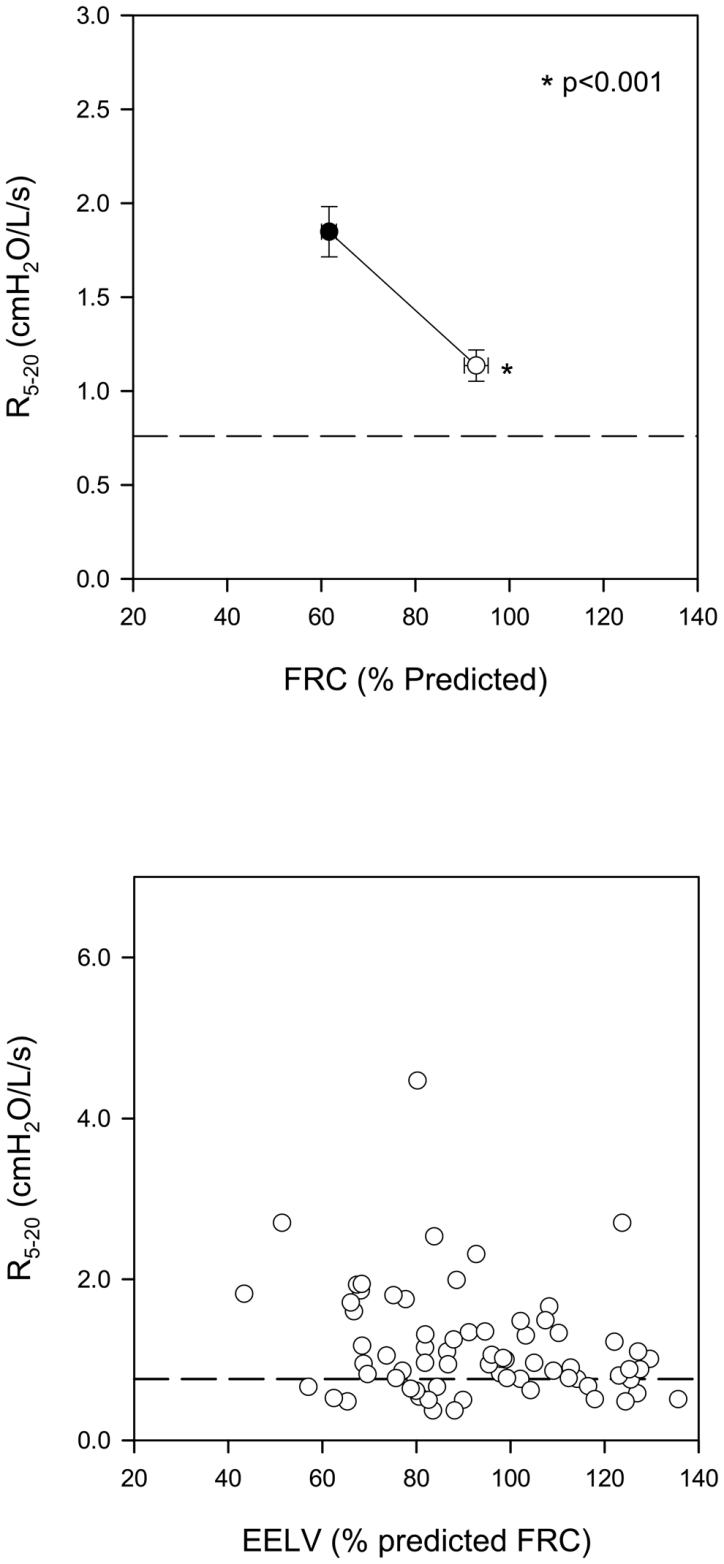
Top Panel: R_5−20_ at baseline and during voluntary inflation. Data are mean ± SE. Dotted line represents the upper limit of normal. Bottom Panel: Individual values obtained during voluntary inflation are plotted as a function of the absolute EELV at which it was obtained. EELV is expressed as % predicted FRC. Data are shown for 50/71 patients in whom maneuvers were acceptable for analysis. Dotted line represents the upper limit of normal. (• = baseline ○ = voluntary inflation).

The effect of voluntary inflation on R_5−20_ is illustrated in Figure 5. The top panel illustrates elevated baseline R_5−20_ (1.89±0.13 cmH_2_O/l/s). Significant improvement was noted upon voluntary inflation; however, the mean value remained elevated (1.16±0.8 cmH_2_O/l/s, p<0.001). The bottom panel relates individual data to the EELV achieved during voluntary inflation, expressed as % predicted FRC. Persistent abnormality in R_5−20_ was noted in the majority of individuals. When analysis was limited to subjects with EELV >80% of the predicted FRC, R_5−20_ remained abnormal in 68% of subjects. In the remaining 32%, R_5−20_ returned to normal indicating that the baseline abnormality was attributable to reduced FRC in those subjects.

**Figure 6:**
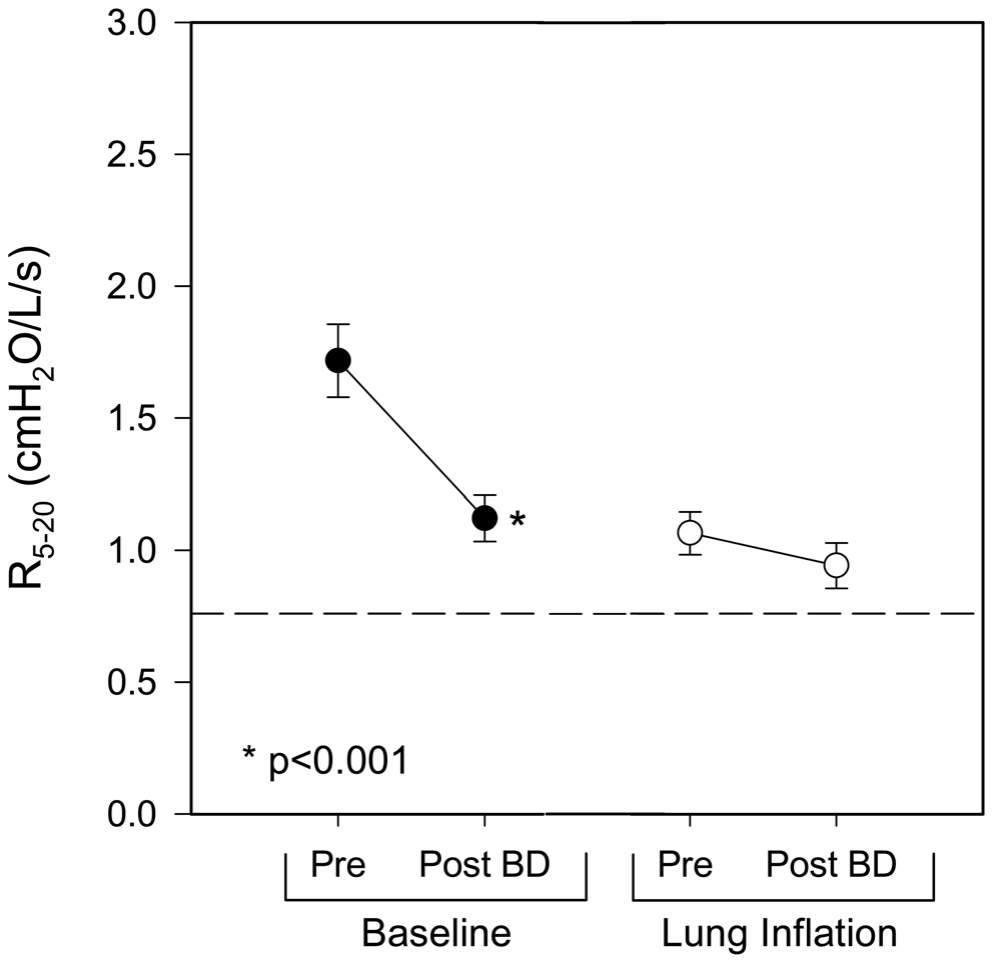
Effect of bronchodilator administration on R_5−20_ at baseline and during voluntary inflation to predicted FRC. Data are mean ± SE. Dotted line represents the upper limit of normal. (• = baseline ○ = voluntary inflation).

Figure 6 illustrates the effect of bronchodilator on baseline and residual abnormality of R_5−20_ at baseline and during voluntary inflation. At baseline, a 35% reduction in mean R_5−20_ was noted following bronchodilator administration (1.72±0.14 vs. 1.12±0.09 cmH_2_O/l/s, p<0.001). In contrast, during voluntary inflation, no broncho-reactivity was observed (1.07±0.08 vs. 0.94±0.09 cmH_2_O/l/s, p = ns) and residual abnormality of R_5−20_ persisted.

## Discussion

The present study evaluated the inter-relationship between resting lung volume and respiratory function in obese subjects with no clinical evidence of cardio-pulmonary disease. Lung dysfunction was characterized by reduction in FRC with associated airway compression. Despite normal FEV_1_/VC, there was evidence of respiratory dysfunction based on oscillometric parameters. Subjects demonstrated increased respiratory system resistance (R_20_) with frequency dependence (R_5−20_) and associated bronchodilator responsiveness. The abnormality in respiratory resistance was volume dependent since specific conductance (SG_rs_ @ 20 Hz) was normal and remained unchanged during voluntary inflation. In contrast, frequency dependence of resistance did not consistently return to normal during voluntary inflation even in subjects that achieved an EELV similar to their predicted FRC. When frequency dependence of resistance normalized during voluntary inflation, mass loading was considered the predominant abnormality; in contrast, when residual abnormality was demonstrable, mechanisms for respiratory dysfunction in addition to mass loading could be invoked.

The observed lung volume abnormalities are in accord with previous publications of obesity and characterized by decreased FRC and ERV, with TLC usually within normal limits. [3], [4], [8], [36] In the present study, reduction in ERV was associated with abnormal R_5−20_, compatible with heterogeneity of distal respiratory mechanics.[17]–[19], [37] This relationship is in accord with curtailment of ERV in obesity as a dynamic phenomenon due to progressive distal airway narrowing upon exhalation generating a pattern of “expiratory restriction”.

A “true restrictive” pattern with TLC below 80% predicted was observed in a subset of subjects and is in accord with previous observations in obesity. [2], [4], [8] TLC was normal in the majority of subjects despite reduction in FRC, since IC was elevated to supernormal values. However, reduced TLC was observed in subjects who failed to increase IC to supernormal values, shifting the pattern from an expiratory restrictive dysfunction to a global reduction of both inspiratory and expiratory function. Potentially, reduction in TLC in obese subjects results from an imbalance between inspiratory muscle strength and the elastic load imposed on the respiratory system in the presence of reduced resting lung volume. When IC, maximal inspiratory pressure and respiratory elastance (X_5_) were considered in a multivariate analysis (data not shown), subjects who did not achieve supernormal IC tended to have more abnormal elastance and lower maximal inspiratory pressure.

Addition of oscillometry to assessment of pulmonary function in obesity revealed abnormalities in resistance and frequency dependence of resistance despite normal airflow as assessed by spirometry. This suggests that either spirometry is not sensitive enough to detect respiratory abnormalities in obesity or that these abnormalities are located more peripherally and therefore not detectable by spirometry (lung “silent zone”). [11] Abnormalities were evident in essentially all subjects, thus confounding the ability of oscillometry to detect associated respiratory dysfunction independent of the effects of mass loading.

The relation of lung volume to resistance has been demonstrated with varying degrees of obesity by calculation of Gaw from plethysmographic resistance.[12]–[15] In the present study, the proportionality of the increase in resistance to the decrease in resting lung volume was addressed by referencing R_20_ to the lung volume at which it was measured for calculation of specific conductance in each individual subject. Specific conductance was calculated utilizing R_20_ (SG_rs_ @ 20 H_Z_) since this laboratory has previously shown that R_20_ is linearly related to pulmonary resistance (R_L_) measured by esophageal manometry and is minimally affected by respiratory system heterogeneity. [29] The clinical utility of conductance calculated from oscillometric parameters has been established by demonstrating that subjects with documented asthma had abnormally low values for conductance compared to normal controls. [38] Since specific conductance was within normal limits at baseline and remained unchanged upon lung inflation, the increase in R_20_ appeared to be fully explained by the decrease in lung volume in these subjects.

Frequency dependence of resistance was also detected in these obese subjects. This oscillometric parameter has been generally attributed to non-uniformity of airflow distribution, which may reflect regional functional abnormalities in the distal lung.[17], [19], [26]–[29] In obesity, distal airway dysfunction may occur due to either mass loading induced airway compression and/or intrinsic airway disease. In the present study, the relative contribution of these abnormalities to presence of frequency dependence of resistance cannot be determined. However, frequency dependence improved following voluntary inflation to predicted FRC, compatible with a more homogeneous lung from opening of previously compressed airways. Residual frequency dependence of resistance was still present in many subjects suggesting potential for intrinsic airway disease/inflammation. In either case, the presence of respiratory dysfunction at baseline FRC may contribute to respiratory symptoms and gas exchange abnormalities that remain unexplained in obese subjects who present with normal airflow on spirometry.

Multiple confounders need to be considered when interpreting these results. Persistent frequency dependence of resistance could reflect failure of the inflation maneuver to reach predicted FRC; however, residual abnormality was evident even in the majority of subjects who increased EELV to predicted FRC. A prolonged period of lung inflation may have been required to fully reverse the abnormality, but this could not be assessed due to the voluntary nature of the maneuver. Additionally, residual abnormality may relate to increased chest wall impedance produced by sustained inspiratory muscle activity. This factor would lead to increased frequency dependence of resistance as chest wall tension reaches its maximum value near TLC. In the present study, a more modest degree of inflation to predicted FRC resulted in a decrease in frequency dependence indicating that on balance the opening of distal airways was the predominant effect of the inflation maneuver.

Bronchodilator responsiveness of frequency dependence of resistance was demonstrated at baseline but was not evident following voluntary inflation to predicted FRC. Multiple mechanical factors have been invoked to explain the interaction between airway reactivity and lung volume that are potentially applicable to the reduced FRC in obesity. [15], [39] Prior studies have confirmed that airway reactivity can be modulated by alterations in lung volume by either deep inspiration and/or artificial reduction of FRC to simulate obesity. [15], [40], [41] These considerations support that compression of airways from reduction of FRC was the mechanism responsible for altered airway reactivity. However, lack of bronchodilator responsiveness of the residual abnormality at the restored lung volume suggests potential intrinsic airway dysfunction independent of the effects of reduced resting lung volume. The residual abnormality in R_5−20_ suggests distal airway dysfunction that may relate to residual bronchial hyperreactivity that has been observed in response to methacholine post weight loss despite normalization of resting lung volume. [42] Of note, the present study included data mostly of female subjects in accord with the population concurring to the Bellevue Hospital Bariatric Center therefore if gender differences exist in the effects of restoration of lung volume on airway responsiveness, they could not be identified in the present study. [15].

In summary, the present study demonstrates that despite normal airway function by spirometry abnormalities in resistance and distal heterogeneity are detected by oscillometry in obese subjects. Lung volume reduction from mass loading of the respiratory system is the main contributory mechanism for these findings. However, abnormalities in distal heterogeneity are uncovered once lung volume is restored indicating that pathological processes may be masked at baseline by the effects of mass loading with consequent airway compression. Multiple conditions may be responsible for these abnormalities including vascular congestion, airway inflammation related to metabolic syndrome, and/or overlap with coexisting diseases such as asthma, and presumably would vary in individual patients.
